# Increasing Antimicrobial Resistance in Nontyphoidal Salmonella Isolates in Australia from 1979 to 2015

**DOI:** 10.1128/AAC.02012-17

**Published:** 2018-01-25

**Authors:** Deborah A. Williamson, Courtney R. Lane, Marion Easton, Mary Valcanis, Janet Strachan, Mark G. Veitch, Martyn D. Kirk, Benjamin P. Howden

**Affiliations:** a Microbiological Diagnostic Unit Public Health Laboratory, Department of Microbiology & Immunology, The University of Melbourne at The Doherty Institute for Infection and Immunity, Melbourne, Australia; b Department of Health and Human Services, Victoria, Australia; c Department of Health and Human Services, Tasmania, Australia; d Australian National University, Canberra, Australia

**Keywords:** Salmonella enterica, epidemiology, antimicrobial resistance, public health, Salmonella, surveillance studies, zoonotic infections

## Abstract

Australia has high and increasing rates of salmonellosis. To date, the serovar distribution and associated antimicrobial resistance (AMR) patterns of nontyphoidal Salmonella enterica (NTS) in Australia have not been assessed. Such information provides critical knowledge about AMR in the food chain and informs decisions about public health. We reviewed longitudinal data on NTS in two Australian states over a 37-year period, between 1979 and 2015, and antimicrobial resistance since 1984. Overall, 17% of isolates were nonsusceptible to at least one antimicrobial, 4.9% were nonsusceptible to ciprofloxacin, and 0.6% were nonsusceptible to cefotaxime. In total, 2.5% of isolates were from invasive infections, with no significant difference in AMR profiles between invasive and noninvasive isolates. Most isolates with clinically relevant AMR profiles were associated with travel, particularly to Southeast Asia, with multiple “incursions” of virulent and resistant clones into Australia. Our findings represent the largest longitudinal surveillance system for NTS in Australia and provide valuable public health knowledge on the trends and distribution of AMR in NTS. Ongoing surveillance is critical to identify local emergence of resistant isolates.

## INTRODUCTION

Salmonella enterica is a major pathogen of humans and animals and can be broadly divided into human-restricted typhoidal Salmonella (serovars Typhi and Paratyphi A, B, and C) and zoonotic nontyphoidal Salmonella (NTS), which includes all other S. enterica serovars (1, 2). Unlike typhoidal Salmonella, NTS serovars differ widely in their host reservoirs and disease manifestations. Although most NTS serovars are associated with self-limiting gastroenteritis, extraintestinal invasive disease is well recognized, particularly with specific serovars such as. *S*. Choleraesuis. Moreover, in certain vulnerable populations such as infants, the elderly, and immunocompromised patients, invasive NTS is associated with severe infection, and complications may result in death, even in developed countries (1–3). Therefore, effective antimicrobial treatment of invasive NTS infections is critical (1).

Over the past 2 decades, there have been increasing reports of antimicrobial resistance (AMR) in NTS to a range of clinically important antimicrobial classes, particularly fluoroquinolones and extended-spectrum cephalosporins (4, 5), prompting both the World Health Organization and the Centers for Disease Prevention and Control to deem AMR in NTS a major threat to public health (6, 7). Rates of resistance among NTS vary depending on locally important serovars in different geographic regions, with some serovars (e.g., *S*. Kentucky and *S*. Typhimurium DT104) emerging as important multiresistant and globally disseminated clones (8, 9). Emerging AMR in nonhuman NTS isolates also poses a significant health threat, particularly given the zoonotic transmission of NTS and the global nature of the food production supply chain.

Compared to several other industrialized countries, Australia has relatively high rates of notified salmonellosis. For example, in 2015, the notification rate for salmonellosis in Australia was 71.7 per 100,000 population (10), compared to a laboratory notification rate of 15.2 per 100,000 population in the United States in 2013 and a laboratory notification rate of 14.8 per 100,000 population in England and Wales in 2015 (11, 12). In Australia, there are unique characteristics relevant to enteric disease, including climate, biodiversity, relative geographic isolation, and an extensive livestock population. Despite high rates of salmonellosis, the trends and prevalence of AMR in NTS in Australia have never been systematically assessed. Such information may provide valuable knowledge on the dissemination of AMR in the food chain and allow informed decisions about surveillance requirements and public health interventions. Here, using longitudinal surveillance data, we describe the serovar distribution, invasive potential, and AMR profiles of NTS isolates from humans in Australia, specifically from the states of Victoria and Tasmania. To our knowledge, these data represent the most continuous and systematically collected available data on serotype distribution and AMR in NTS in Australia and highlight the need for comprehensive surveillance of AMR in enteric pathogens.

## RESULTS

### Longitudinal distribution of NTS serovars.

**FIG 1:**
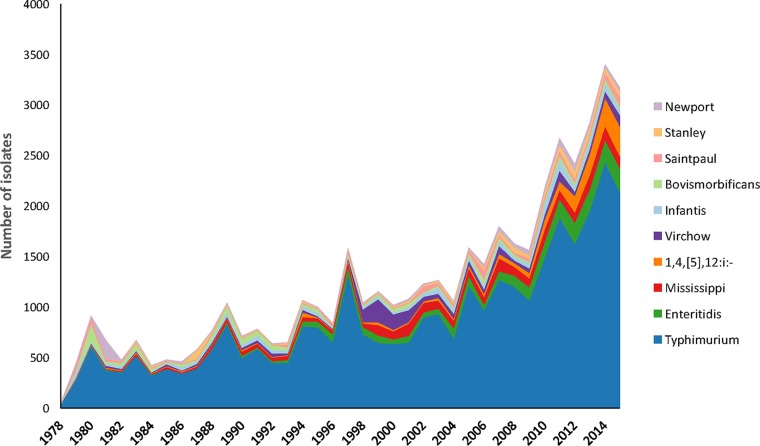
Number and distribution of the 10 most-common nontyphoidal Salmonella serovars identified through the National Enteric Pathogen Surveillance Scheme, Victoria and Tasmania, Australia, 1979 to 2015.

Between 1979 and 2015, a total of 58,830 isolates were obtained from Victoria (53,317; 91%) and Tasmania (5,513; 9%) (see Data Set S1 in the supplemental material). The most-common serovar over the study period was *S*. Typhimurium, accounting for 56.2% (33,081/58,830) of all NTS isolates (57.9% [30,892/53,317] of Victorian isolates and 39.7% [2,189/5,513] of Tasmanian isolates). Together, the 10 most-common serovars accounted for 78.1% (45,932/58,830) of all NTS, and their distribution is illustrated in Fig. 1. Prior to 1994, *S*. Bovismorbificans was the second most-common serovar in Victoria (5.2% [644/12,485]), replaced between 1994 and 2013 with *S*. Enteritidis (5.4% [1,817/33,541]) and since 2014 by a monophasic variant of *S*. Typhimurium with the antigenic formula 1,4,[5],12:i:− (Salmonella 1,4,[5],12:i:−) (6.1% [448/7,291]). In Tasmania, the second most-common serovar was *S*. Mississippi, where it accounted for 37.2% (2,048/5,513) of all NTS in Tasmania, compared to only 0.4% (201/53,317) of all NTS in Victoria.

### Trends in clinically important antimicrobial resistance and association with specific serovars.

**FIG 2:**
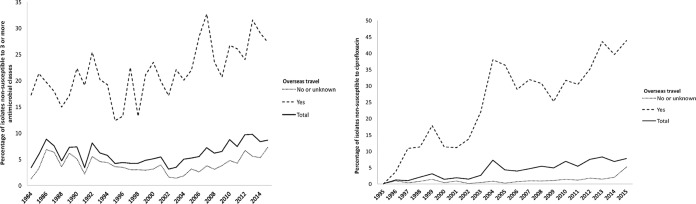
Prevalence of multidrug resistance (1984 to 2015) (left) and ciprofloxacin nonsusceptibility (1995 to 2015) (right) in patients with nontyphoidal Salmonella enterica, with and without recent overseas travel.

A total of 54,451 NTS isolates from humans identified between 1984 and 1995 underwent antimicrobial susceptibility testing. Overall, 17.4% (9,482/54,451) of all isolates were resistant to at least one antimicrobial. However, the prevalence of multidrug resistance (MDR; defined as nonsusceptibility to at least one agent in three or more classes of antimicrobials) was generally low, ranging from 3.4% (22/650) in 1984 to a high of 9.7% (336/3,452) in 2013 (Fig. 2, left). In seven serovars, the prevalence of MDR was greater than 30% (*S*. Panama, *S*. Blockley, Salmonella 1,4,[5],12:i:−, *S*. Kentucky, *S*. Rissen, *S*. Schwarzengrund, and *S*. Corvallis) (Table 1). A further nine serovars were significantly associated with MDR (see Data Set S2 in the supplemental material). MDR isolates were significantly more likely to be found in patients reporting overseas travel or residence (odds ratio [OR], 7.07; 95% confidence interval [CI], 6.58 to 7.59; *P* < 0.001), with a temporal increase in the prevalence of MDR from 12.4% in 1995 to 27.3% in 2015 among travel-associated isolates (Fig. 2, left). Thailand (29.7% [471/1,586]) and Vietnam (8.8% [139/1,1,586]) were the most commonly reported overseas locations for patients with MDR infections.

**TABLE 1:** Nontyphoidal Salmonella enterica serovars associated with clinically relevant antimicrobial resistance

Antimicrobial resistance class and Salmonella serovar	No. (%) of nonsusceptible/total no. of isolates	Adjusted^*a*^ OR (95% CI)	*P* value
Multidrug resistance^*b*^ (1984-2015)			
Panama	108/142 (76.1)	46.9 (31.7–69.5)	<0.001
Blockley	104/140 (74.3)	51.3 (34.8–75.7)	<0.001
Kentucky	76/106 (71.7)	33.5 (21.8–51.5)	<0.001
Rissen	68/107 (63.6)	23.7 (15.9–35.4)	<0.001
Schwarzengrund	72/114 (63.2)	21.8 (14.8–32.2)	<0.001
Salmonella 4,[5],12:i:−	588/801 (73.4)	48.9 (41.1–58.2)	<0.001
Corvallis	101/240 (42.1)	9.2 (7.1–12)	<0.001
All *S*. enterica serovars	3,615/54,451 (6.64)		
Ciprofloxacin nonsusceptible (1994-2015)			
Schwarzengrund	81/102 (79.4)	74.6 (44.6–124.9)	<0.001
Corvallis	180/240 (75.0)	51.6 (38.3–69.6)	<0.001
Kentucky	59/80 (73.8)	55.6 (32.3–95.7)	<0.001
Blockley	27/38 (71.1)	173.2 (75.5–397.2)	<0.001
Hadar	155/309 (50.2)	67.1 (49.6–90.8)	<0.001
Enteritidis	848/2,406 (35.2)	17.1 (15.5–19.0)	<0.001
Braenderup	35/125 (28.0)	6.5 (4.3–9.6)	<0.001
Give	24/96 (25.0)	7.8 (4.8–12.6)	<0.001
Rissen	14/98 (14.3)	2.8 (1.6–5.1)	<0.001
Agona	56/408 (13.7)	3.6 (2.7–4.9)	<0.001
Stanley	62/654 (9.5)	1.8 (1.4–2.4)	<0.001
Montevideo	14/172 (8.1)	1.8 (1.1–3.2)	0.032
Virchow	76/1,509 (8.1)	1.8 (1.1–3.2)	0.049
All S. enterica isolates	2,197/45,071 (4.9)		
Cephalosporin nonsusceptible (2001-2015)			
Salmonella 4,[5],12:i:−	40/800 (5.0)	7.9 (5.5–11.5)	<0.001
Corvallis	7/240 (2.9)	5.1 (2.4–11)	<0.001
Rissen	2/84 (2.4)	3.7 (0.9–15.1)	0.072
Stanley	11/577 (1.9)	3.3 (1.8–6.1)	<0.001
Saintpaul	8/626 (1.3)	2.4 (1.2–5)	0.015
All S. enterica isolates	204/34,294 (0.6)		

a Adjusted for year.

b Nonsusceptible to three or more classes of antimicrobials; serovars with MDR prevalence of >30% are listed.

Ciprofloxacin susceptibility testing commenced routinely in 1994, and nonsusceptibility increased steadily from 0.1% (1/1,243) in 1995 to 7.8% (293/3,783) in 2015, with a median annual increase of 0.7% (Fig. 2, right). Ciprofloxacin nonsusceptibility was significantly more common in isolates from patients who reported overseas travel or residence than in patients who did not (29.7% versus 1.4%, *P* < 0.001). Four NTS serovars had a prevalence of ciprofloxacin nonsusceptibility of greater than 70% (*S*. Schwarzengrund, *S*. Corvallis, *S*. Kentucky, and *S*. Blockley), and a further nine serovars were significantly associated with ciprofloxacin nonsusceptibility (Table 1). Of the patients with fluoroquinolone-nonsusceptible *S*. Schwarzengrund, 73/81 (90.1%) had a reported history of overseas travel. Similarly, fluoroquinolone-nonsusceptible *S*. Corvallis (82.8% [149/180]), *S*. Kentucky (72.9% [43/59]), and *S*. Blockley (88.9% [24/27]) were also associated with overseas travel. When available, the most commonly reported overseas destinations were Thailand (46/289; 15.9%), Malaysia (41/289; 14.2%), and Indonesia (28/289; 9.7%).

Nonsusceptibility to third-generation cephalosporins remained low at 0.6% (204/34,498), ranging from 0.1% (1/1,516) upon commencement of routine testing in 2002 to 1.0% (38/3,782) in 2015. Cephalosporin nonsusceptibility was significantly associated with five serovars: Salmonella 1,4,[5],12:i:−, *S*. Corvallis, *S*. Rissen, *S*. Stanley, and *S*. Saintpaul (Table 1). Overseas travel or residence was commonly reported among patients with *S*. Corvallis (192/240; 80.0%), *S*. Rissen (79/107; 73.8%), and *S*. Stanley (435/830; 52.4%) isolates. The prevalence of azithromycin resistance was low (17/1,855 isolates tested; 0.9%); no carbapenem resistance was detected.

### Associations with invasive NTS infection.

Overall, 1,461/58,420 isolates (2.5%) were associated with invasive disease, of which 897/1,455 (61.7%) were from males. Males were significantly more likely to have invasive disease (OR, 1.70; 95% CI, 1.52 to 1.88; *P* < 0.001). The median age of patients with invasive isolates was 51 years (interquartile range [IQR], 22 to 71 years) and was significantly higher than the median age of patients with noninvasive isolates (22 years; IQR, 4 to 42 years). A total of 13 serotypes (including *S*. Enteritidis and *S*. Mississippi) were associated with a significantly higher proportion of invasive disease than *S*. Typhimurium (Table 2). However, the absolute number of invasive cases was highest for *S*. Typhimurium (639 cases), followed by *S*. Virchow (166 cases) and then *S*. Enteritidis (118 cases). The serovar with the highest proportion of invasive isolates was *S*. Dublin (36.6%), followed by *S*. Panama (27.0%) and then *S*. Virchow (9.5%). A small, nonsignificant increase in the proportion of invasive isolates over time was observed (0.7% per year; 95% CI, 0.1% to 1.5%; *P* = 0.055). A total of 271/1,417 (20.0%) invasive isolates identified since 1984 were resistant to at least one clinically relevant antimicrobial agent. However, isolates from bloodstream infections were not significantly more resistant than similar isolates from stool (Table 3).

**TABLE 2:** Nontyphoidal Salmonella enterica serovars associated with invasive disease, 1979 to 2015, Australia

Salmonella serovar^*a*^	Total no.	No. (%) of isolates associated with invasive disease	Relative risk of invasiveness compared to *S*. Typhimurium (95%CI)	*P* value
Dublin	101	37 (36.3)	18.8 (14.4–24.6)	<0.001
Panama	148	40 (26.8)	13.9 (10.6–18.3)	<0.001
Virchow	1,740	166 (9.5)	4.9 (4.2–5.8)	<0.001
Schwarzengrund	146	12 (8.2)	4.2 (2.4–7.3)	<0.001
Heidelberg	386	31 (7.9)	4.1 (2.9–5.8)	<0.001
Javiana	126	9 (7.0)	3.7 (1.9–6.9)	<0.001
Bredeney	105	6 (5.7)	2.9 (1.3–6.4)	0.007
Thompson	386	6 (5.5)	2.9 (1.3–6.4)	<0.001
Chester	240	19 (4.9)	2.5 (1.6–4.0)	0.046
Enteritidis	1,299	118 (4.6)	2.4 (2.0–2.9)	<0.001
Corvallis	2,228	9 (3.8)	1.9 (1.0–3.7)	0.031
Bovismorbificans	101	45 (3.4)	1.8 (1.3–2.4)	<0.001
Mississippi	148	58 (2.6)	1.3 (1.0–1.7)	<0.001
Typhimurium	33,081	639 (1.9)	NA^*b*^	NA

a Only serovars with >100 isolates were included in the analysis.

b NA, not applicable (because *S*. Typhimurium is the reference isolate).

**TABLE 3:** Antimicrobial resistance of nontyphoidal Salmonella enterica blood and stool isolates to clinically relevant antimicrobials, by serotype, 1984 to 2015^*a*^

Salmonella serovar	Isolates from blood and stool		Isolates from blood		Isolates from stool		Odds of resistance to ≥1 antimicrobial^*b*^ in blood compared to stool	
	*n*	No. (%) resistant to ≥1 antimicrobial	*n*	No. (%) resistant to ≥1 antimicrobial	*n*	No. (%) resistant to ≥1 antimicrobial	OR (95% CI)	*P* value
Typhimurium	30,890	3,502 (11.3)	543	75 (13.8)	29,682	3,342 (11.2)	1.3 (1.0–1.6)	0.063
Virchow	1,700	274 (16.1)	148	21 (14.1)	1,474	246 (16.6)	0.8 (0.5–1.3)	0.435
Enteritidis	2,530	1,298 (51.3)	108	54 (50.0)	2,340	1,217 (52.0)	0.9 (0.6–1.4)	0.683
Mississippi	2,205	28 (1.2)	52	0 (0.0)	2,015	27 (1.3)	Undefined	
Panama	142	111 (78.1)	39	34 (87.1)	98	73 (74.4)	2.3 (0.8–6.6)	0.112
Bovismorbificans	1,001	112 (11.1)	32	5 (15.6)	928	99 (10.6)	1.6 (0.6–4.1)	0.379
Dublin	92	8 (8.6)	30	5 (16.6)	47	2 (4.2)	4.5 (0.8–24.9)	0.085
Heidelberg	381	100 (26.2)	30	7 (23.3)	339	90 (26.5)	0.8 (0.4–2.0)	0.702
Infantis	1,329	108 (8.1)	20	0 (0.0)	1,234	97 (7.8)	Undefined	
Saintpaul	892	103 (11.5)	18	2 (11.1)	830	96 (11.5)	1.0 (0.2–4.2)	0.952

a Serovars listed are the 10 most-common isolates from blood; *n*, total number of isolates in the category; OR, odds ratio; 95% CI, 95% confidence interval.

b Resistance “to ≥1 antimicrobial” refers to tested antimicrobials.

## DISCUSSION

In this study, we demonstrate an increasing incidence of multidrug-resistant Salmonella in southeastern Australia over time, related mainly to travel overseas. Moreover, we observed key differences between the serotype distributions of salmonellosis in Australia and other developed regions. Across the study period, the ratio of *S*. Typhimurium to *S*. Enteritidis isolation was approximately 13:1. This ratio is in marked contrast to observations from Europe and North America, where *S*. Enteritidis is generally more prevalent and where *S*. Enteritidis infections are strongly associated with poultry sources (13–15). A 2013 study from the European Food Safety Authority (EFSA) and the European Center for Disease Prevention and Control (ECDC) observed that *S*. Enteritidis and *S*. Typhimurium represented 39.5% and 20.2%, respectively, of serovars in human cases from 25 member states (16). Importantly, *S*. Enteritidis is not considered endemic to Australian layer flocks, and most *S*. Enteritidis infections in Australia are related to overseas travel (17). In Australia, between 2001 and 2011, there was a significant increase in egg-associated salmonellosis outbreaks, with *S*. Typhimurium implicated in over 90% of such outbreaks, suggesting a major role for *S*. Typhimurium in egg-associated salmonellosis in Australia (17). Another notable finding was that *S*. Mississippi was the second most-common serovar in Tasmania, accounting for 37.2% of all NTS in Tasmania, compared to <1% in Victoria. *S*. Mississippi is an uncommon serovar in several other regions, accounting for only 1.1% of NTS infections in the United States in 2013 (18). A previous case-control study between October 2001 and December 2002 suggested that indirect contact (via contaminated land or water environments) with Australian native wildlife may be an important reservoir for this serotype in Tasmania (19). Given the high prevalence of *S*. Mississippi in human NTS infections in Tasmania, further work should investigate the environmental niches and transmission of this serovar.

We also defined the trends in AMR over 3 decades and found that the prevalence of clinically relevant AMR in NTS isolates in Australia was generally comparable to that of other industrialized countries. For example, a 2014 EFSA and ECDC report assessing AMR in 14,412 NTS isolates from 22 European countries observed nonsusceptibility in 1.1% of isolates for cefotaxime and 8.8% of isolates for ciprofloxacin (compared to 1.0% and 6.9%, respectively, in our study in 2014) (20). Fluoroquinolones have never been approved for use in food-producing animals in Australia (21). However, given our observed association between overseas travel and ciprofloxacin nonsusceptibility, it is highly likely that importation of resistant clones is largely responsible for the relatively high rates of ciprofloxacin resistance among NTS isolates in Australia. Supporting this theory is the finding that ciprofloxacin resistance was strongly associated with known internationally circulating AMR serovars, particularly *S*. Corvallis, *S*. Kentucky, and *S*. Schwarzengrund. For example, fluoroquinolone-resistant *S*. Corvallis has been described from patients in the Netherlands and Denmark and from beef, pork, and chicken meat in Thailand (22, 23). Similarly, *S*. Kentucky sequence type 198 (ST198) has been reported globally as a highly resistant serovar (8). One limitation of our study is that geographical associations of specific serovars may be biased by the preferred destinations of Australian travelers and migrant populations within Australia. Future work should confirm our findings with augmented epidemiological surveillance.

An association between specific NTS serovars and invasive salmonellosis has previously been described (3), although there is geographic variation in the prevalence of invasiveness among particular serovars (24). We investigated this association among Victorian and Tasmanian NTS isolates and identified *S*. Dublin and *S*. Panama as having strong associations with invasiveness. Our findings are broadly in keeping with work from other geographic regions that described these serovars as particularly invasive (3, 24), although to date, there is little understanding of the host and/or bacterial factors that underlie this association. We also observed that both *S*. Virchow and *S*. Enteritidis had a significantly higher association with invasiveness than *S*. Typhimurium (9.5% and 4.6% versus 1.9%, respectively; *P* < 0.001). Unlike a similar recent study from the United States (25), we did not identify an association between bloodstream isolates and increased AMR. The reason for this difference is unclear but may relate to the low prevalence of resistance in *S*. Typhimurium, the most-common cause of invasive and noninvasive infections in our study. In contrast, Angelo et al. (25) found that 53% and 36% of *S*. Typhimurium blood and stool isolates, respectively, in the United States were resistant to at least one clinically relevant antimicrobial, compared to 10.1% and 9.3% in our study. It is possible that the higher overall rates of resistance in the study by Angelo et al. may have contributed to failure of empirical therapy, with resistant isolates more likely to be observed in bacteremia.

Collectively, our findings show that resistance to clinically important antimicrobials is largely associated with overseas travel, while most “endemic” locally acquired NTS strains remain susceptible. This observation has important implications for the treatment and control of resistant NTS in Australia. For example, clinicians should consider travel-related risk factors when determining if and what empirical antimicrobial therapy may be appropriate to treat salmonellosis. Moreover, in an increasing era of culture-independent diagnostic testing for enteric pathogens (26), our results highlight the necessity for ongoing culture and antimicrobial susceptibility testing to monitor resistance trends, particularly in patients recently returned from overseas.

In summary, our work provides the first systematic overview of the trends and associated AMR patterns of NTS in Australia. At present, Australia does not have a comprehensive national surveillance scheme for continually monitoring AMR in NTS isolates. Our findings represent the largest longitudinal surveillance system for NTS in Australia and provide valuable public health knowledge on the trends and distribution of AMR in NTS. It is vital to strengthen this platform for ongoing and enhanced surveillance, which is critical in the current era of rapidly increasing antimicrobial resistance.

## MATERIALS AND METHODS

### Setting and data sources.

In Australia, the National Enteric Pathogens Surveillance Scheme (NEPSS) is a dedicated surveillance system for human and nonhuman enteric pathogens (including Salmonella) that has been operated by the Microbiological Diagnostic Unit Public Health Laboratory (MDU PHL) at the University of Melbourne since 1978. Data in NEPSS include (i) epidemiological typing data such as serotype, phage type for selected serovars, and more recently, multiple-locus variable number of tandem repeats analysis profiles for Salmonella Typhimurium, (ii) antimicrobial susceptibility data, and (iii) basic demographic data for human cases (e.g., age, gender, state of residence). When provided, travel history for patients with salmonellosis is also recorded in NEPSS. All NTS isolates from the states of Victoria and Tasmania are referred to MDU PHL; however, submission to NEPSS is voluntary for other Australian states and territories. Therefore, to provide continuous and substantially complete data, we specifically analyzed data from Victoria and Tasmania. Data on the distribution of NTS serovars in Victoria and Tasmania were obtained from the NEPSS database from the period of 1 January 1 1979 to 31 December 2015, and data on AMR were obtained for the period of 1 January 1984 to 31 December 2015 (following commencement of comprehensive routine susceptibility testing). To assess the completeness of data in NEPSS, we compared the number of NTS isolates received through NEPSS with the number of notifications for salmonellosis in the Australian National Notifiable Diseases Surveillance Scheme (NNDSS), which has notification data for nontyphoidal salmonellosis from each Australian state and territory from 1991 onwards (available at http://www9.health.gov.au/cda/source/cda-index.cfm). Overall, the number of notifications for NTS in NEPSS closely matched the notifications in NNDSS (see Fig. S1 in the supplemental material).

Salmonella isolates were defined as “invasive” if they were isolated from normally sterile body sites, namely, blood, cerebrospinal fluid (CSF), peritoneal fluid, pleural fluid, bone, or joint. Isolates from wounds or urine were classified as noninvasive, as clinical data were not available to allow differentiation between colonization and infection.

### Identification and antimicrobial susceptibility testing.

Identification, epidemiological typing, and antimicrobial susceptibility testing (AST) of all NTS included in this study were performed at MDU PHL. All Salmonella isolates were subjected to serotyping according to the Kauffmann-White scheme (27). AST on all NTS was performed using agar breakpoint dilution, generating qualitative categorical AST data (breakpoint concentrations are provided in Data Set S3 in the supplemental material). Routine testing for the following agents was performed from 1984 through 2015: ampicillin, chloramphenicol, kanamycin, nalidixic acid, streptomycin, sulfathiazole, trimethoprim, and tetracycline. Ciprofloxacin was tested routinely from 1994 onwards, gentamicin from 2000 onwards, cefotaxime from 2001, and azithromycin and meropenem from 2015. Isolates displaying intermediate resistance were classified as nonsusceptible. Multidrug resistance (MDR) was defined as nonsusceptibility to at least one agent in three or more classes of antimicrobials (28). When possible, contemporaneous Clinical and Laboratory Standards Institute (CLSI) breakpoints were used for interpretation, as described previously (29). There are no CLSI breakpoints for azithromycin resistance in nontyphoidal Salmonella; therefore, in accordance with other Salmonella AMR surveillance systems (25), azithromycin nonsusceptibility was defined as ≥32 μg/ml.

### Statistical analysis.

The chi-square and Fisher exact tests were used to determine differences in the proportion of isolates that were (i) nonsusceptible to each tested antimicrobial class and (ii) nonsusceptible to three or more classes, by year (between 1984 and 2015) and by serovar. Serovars with fewer than 100 total isolates were excluded from serovar-specific analyses. When the proportion of nonsusceptible isolates was greater than the expected value, associations were examined using logistic or negative binomial regression. Covariates were defined *a priori*; regression models with serovar as the dependent variable were adjusted for year of isolation, with all analyses stratified by overseas travel, and those examining year adjusted for serovar. Trends in the proportion of invasive isolates and the three most-prevalent serovars over time were examined using the Poisson regression. All statistical analyses were performed using Stata 13.1 MP, and a two-tailed *P* value of <0.05 was considered significant.

## Supplementary Material

Supplemental material
