# Supplementary material for: Increasing Antimicrobial Resistance in Nontyphoidal Salmonella Isolates in Australia from 1979 to 2015
Source: Antimicrob Agents Chemother. 2018 Jan 25;62(2):e02012-17. doi: 10.1128/AAC.02012-17 (PMC5786757; doi:10.1128/AAC.02012-17)
Supplement: Supplemental material [file supp_62_2_e02012-17__index.html]

Supplemental material 

# Increasing Antimicrobial Resistance in Nontyphoidal Salmonella Isolates in Australia from 1979 to 2015

## Supplemental material

- Supplemental file 1 -

  Fig. S1 and Data Sets S1 to S3

  PDF, 308K
