# Supplementary material for: Increasing Antimicrobial Resistance in Nontyphoidal Salmonella Isolates in Australia from 1979 to 2015
Source: Antimicrob Agents Chemother. 2018 Jan 25;62(2):e02012-17. doi: 10.1128/AAC.02012-17 (PMC5786757; doi:10.1128/AAC.02012-17)
Supplement: Supplemental material [file AAC.02012-17_zac002186879s1.pdf]

SUPPLEMENTARY DATA

Supplementary dataset S1: Serovar distribution
All serovars where n>100 isolates

Salmonella enterica Serovar	Number	% of total isolates
S. Typhimurium	33,081	56.2%
S. Enteritidis	2,554	4.3%
S. Mississippi	2,249	3.8%
S. Virchow	1,755	3.0%
S. Infantis	1,461	2.5%
S. Bovismorbificans	1,309	2.2%
S. Saintpaul	1,066	1.8%
S. Stanley	858	1.5%
Salmonella 1,4,[5],12:i:-	801	1.4%
S. Newport	798	1.4%
S. Agona	611	1.0%
S. Singapore	535	0.9%
S. Hadar	496	0.8%
S. Anatum	460	0.8%
S. Derby	406	0.7%
S. Heidelberg	391	0.7%
S. Chester	388	0.7%
S. Weltevreden	358	0.6%
S. Havana	351	0.6%
S. Mbandaka	336	0.6%
S. Muenchen	329	0.6%
S. Birkenhead	265	0.5%
S. Senftenberg	258	0.4%
S. Montevideo	252	0.4%
S. Corvallis	240	0.4%
S. Oranienburg	230	0.4%
S. Give	209	0.4%
S. Hvittingfoss	208	0.4%
S. Ohio	205	0.3%
S. subsp II ser Sofia	205	0.3%
S. Cerro	194	0.3%
S. Kottbus	189	0.3%
S. Potsdam	177	0.3%
S. Zanzibar	177	0.3%
S. Hessarek	170	0.3%
S. Blockley	168	0.3%
S. Victoria	160	0.3%
S. Bareilly	158	0.3%
S. Panama	149	0.3%
S. London	147	0.2%
S. Kiambu	146	0.2%
S. Schwarzengrund	146	0.2%

S. Braenderup	139	0.2%
S. Adelaide	131	0.2%
S. Javiana	129	0.2%
S. Tennessee	127	0.2%
S. Oslo	124	0.2%
S. Aberdeen	118	0.2%
S. Kentucky	110	0.2%
S. Thompson	110	0.2%
S. Reading	108	0.2%
S. Rissen	107	0.2%
S. Bredeney	106	0.2%
S. Orientalis	105	0.2%
Salmonella 16:l,v:-	105	0.2%
S. Dublin	102	0.2%
Other serovars	2,563	4.4%
**Total**	**58,830**	

Supplementary dataset S2: Serovars associated with critical resistances, 1984-2015

Salmonella serovar	Non-susceptible (n)	Total (n)	%	Adjusted OR[a]	95% CI	P-value
**Multidrug resistance (non-susceptible to three or more classes of antimicrobials**						
S. Blockley	111	168	66.1	51.3	(34.8, 75.7)	<0.001
Salmonella 1,4,[5],12:i:-	588	801	73.4	48.9	(41.1, 58.2)	<0.001
S. Panama	114	149	76.5	46.9	(31.7, 69.5)	<0.001
S. Kentucky	79	110	71.8	33.5	(21.8, 51.5)	<0.001
S. Rissen	68	107	63.6	23.7	(15.9, 35.4)	<0.001
S. Schwarzengrund	72	146	49.3	21.8	(14.8, 32.2)	<0.001
S. Corvallis	101	240	42.1	9.2	(7.1, 12.0)	<0.001
S. Kiambu	40	146	27.4	5.6	(3.8, 8.1)	<0.001
S. Hadar	118	496	23.8	5.3	(4.3, 6.6)	<0.001
S. Stanley	206	858	24.0	4.7	(4.0, 5.6)	<0.001
S. Derby	45	406	11.1	2.6	(1.8, 3.6)	<0.001
S. Give	22	209	10.5	2.4	(1.5, 3.8)	<0.001
S. London	17	147	11.6	2.2	(1.3, 3.6)	0.003
S. Agona	66	611	10.8	2.0	(1.5, 2.6)	<0.001
S. Anatum	50	460	10.9	1.9	(1.4, 2.7)	<0.001
S. Newport	69	798	8.6	1.6	(1.2, 2.1)	<0.001
**Overall**	**3,783**	**58,830**	**6.4**			

[a] logistic regression, adjusted for year (reference serovar, S. Typhimurium)

Supplementary Dataset S3: MIC breakpoint concentrations used in this study

Class	Antimicrobial(s)	Breakpoint concentration (mg/L)
**Penicillin**	Ampicillin	16
**Chloramphenicol**	Chloramphenicol	16
**Folate inhibitors**	Trimethoprim	8
	Sulphathiozole	512
**Fluoroquinolones**	Nalidixic acid	16
	Ciprofloxacin (decreased susceptibility)	0.06
	Ciprofloxacin	2
**Cephalosporins**	Cefotaxime	1
**Tetracycline**	Tetracycline	8
**Aminoglycosides**	Streptomycin	32
	Gentamicin	8
	Kanamycin	16
**Carbapenems**	Meropenem	1

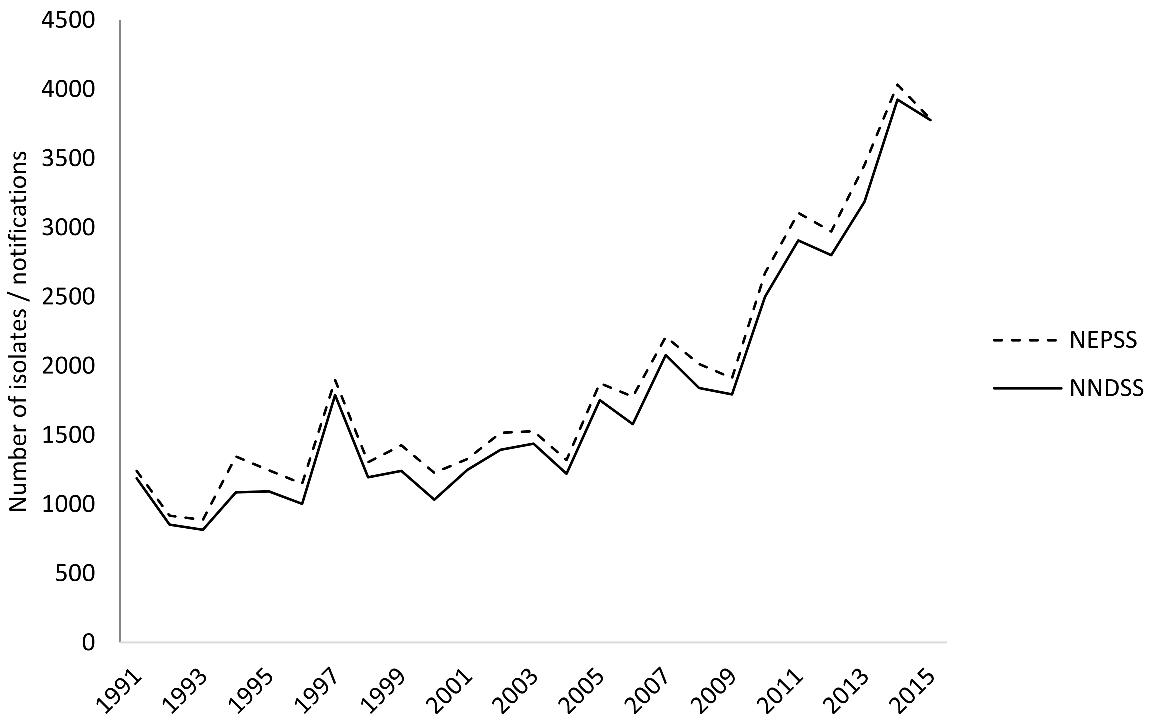

Supplementary Figure 1. Number of non-typhoidal *Salmonella enterica* isolates received through the National Enteric Pathogen Surveillance Scheme (NEPSS) from Victoria and Tasmania (dashed line) and number of notifications for salmonellosis recorded in the National Notifiable Diseases Surveillance System (solid line).
